# The Influence of Branched-Chain Amino Acid Supplementation on Fatigue and Tryptophan Metabolism After Acute and Chronic Exercise in Older Adults: Protocol for a Pilot Randomized Controlled Trial

**DOI:** 10.2196/52199

**Published:** 2023-11-01

**Authors:** Ronna N Robbins, Tiffany Cortes, Jason C O'Connor, Rozmin Jiwani, Monica C Serra

**Affiliations:** 1 Department of Nutrition and Food Science Texas Woman's University Denton, TX United States; 2 South Texas Veterans Health Care System Geriatric Research Education and Clinical Center San Antonio, TX United States; 3 Sam & Ann Barshop Institute for Longevity & Aging Studies Department of Medicine University of Texas Health Health Science Center San Antonio San Antonio, TX United States; 4 South Texas Veterans Health Care System San Antonio, TX United States; 5 Department of Pharmacology University of Texas Health Science Center San Antonio San Antonio, TX United States; 6 School of Nursing University of Texas Health Science Center San Antonio San Antonio, TX United States

**Keywords:** branched-chain amino acids, kynurenine, fatigue, older adults, exercise

## Abstract

**Background:**

Fatigue is a strong predictor of negative health outcomes in older adults. Kynurenine, a metabolite of tryptophan, is strongly associated with fatigue. Reductions in fatigue are observed with exercise; however, exercise training does not completely alleviate symptoms. Branched-chain amino acids (BCAAs) have been shown to have advantageous effects on exercise performance and compete with kynurenine for transport into the central nervous system. Thus, the combination of BCAA and exercise may exert synergized effects of mental and physical fatigue. Therefore, we hypothesize that BCAA added to exercise will shift kynurenine metabolism toward enhanced synthesis of kynurenic acid, thereby reducing fatigue.

**Objective:**

This randomized, double-blind, placebo-controlled trial aims to compare the effects of acute (approximately 45 min) and chronic (8 wk) exercise with and without BCAA supplementation on mental and physical fatigue and assess whether the hypothesized outcomes are modulated by changes in kynurenine metabolism in 30 older adults (n=15, 50% per group).

**Methods:**

Older adults (aged 60-80 y) who do not exercise >2 days per week and self-report fatigue (≥3 on a scale of 1-10) will be recruited. Participants will be randomized to either the exercise+BCAA group or exercise+placebo group. Participants will engage in high-volume, moderate-intensity, whole-body exercise training (aerobic and resistance exercise; either in-person or web-based sessions) 3 times per week for 8 weeks. In addition, participants will consume daily either 100 mg/kg body weight of BCAA (2:1:1 leucine:isoleucine:valine) or placebo (maltodextrin) throughout the 8-week intervention. BCAA and placebo powders will be identical in color and dissolved in 400 mL of water and 2.5 g of a calorie-free water flavor enhancer. Muscle biopsies will be collected before and after the intervention after a 12-hour fast to examine changes in the biomarkers of tryptophan metabolism and inflammation. Our primary outcomes include changes in mental and physical fatigue and metabolism after the 8-week exercise training between the 2 groups. Mental and physical fatigue will be measured before and after the intervention. Mental fatigue will be subjectively assessed through the completion of validated questionnaires. Physical fatigue will be measured by isometric handgrip, 1-repetition maximum, chair rise, 400-meter walk, and cardiopulmonary exercise tests.

**Results:**

The study was funded in March 2022, with an anticipated projected data collection period lasting from January 2023 through December 2023.

**Conclusions:**

The discovery that kynurenine concentrations are associated with fatigue and are responsive to BCAA supplementation during exercise training could have important implications for the development of future interventions, both lifestyle and pharmacologic, to treat fatigue in older adults.

**Trial Registration:**

ClinicalTrials.gov NCT05484661; https://www.clinicaltrials.gov/study/NCT05484661

**International Registered Report Identifier (IRRID):**

DERR1-10.2196/52199

## Introduction

### Background

The prevalence rate of self-reported mental and physical fatigue among older adults is at least 25% in primary care settings [1]. Mental fatigue refers to a decrease in cognitive resources, whereas physical fatigue refers to the inability of muscles to maintain optimal performance. Studies suggest that mental fatigue may be present before the onset of disability and morbidity [2] and has been reported by older adults to be a main reason for activity restriction and poor compliance with exercise interventions [3]. Although prior studies show that both mental and physical fatigue are associated with low-grade inflammation [4], more research is needed to pinpoint specific biological pathways to connect chronic inflammation to fatigue-associated health declines.

One pathway that may play an integral role in fatigue progression is that of the essential amino acid tryptophan. Normally, 90% to 95% of tryptophan is metabolized along the kynurenine pathway in the liver. Inflammation upregulates kynurenine pathway enzymes outside the liver, causing intracellular and circulating kynurenine or oxidative kynurenine metabolite concentrations to rise. The accumulation of kynurenine and its oxidative metabolites are strongly associated with physical fatigue, including weaker grip strength and slower walking speed [5]. Furthermore, they can readily cross into the central nervous system (CNS) and underlie the behavioral and cognitive impairments caused by inflammation [6].

Evidence in animal models suggests that reductions in physical fatigue often observed with exercise training are mediated by skeletal muscle peroxisome proliferator-activated receptor γ coactivator 1α (PGC-1α), inducing a shift of kynurenine to kynurenic acid [7,8]. This is catalyzed by kynurenine aminotransferase (KAT) enzymes, which prevent the oxidative metabolism of kynurenine as well as its entry into the CNS; this may have an influence on mental fatigue. We have shown that reductions in skeletal muscle inflammation are associated with reductions in mental and physical fatigue after exercise training [9]; however, a recent study in older adults also found that exercise training significantly increased skeletal muscle PGC-1α gene expression and KAT isoforms by nearly 2-fold [10]. These data suggest that the tryptophan metabolism pathway may play an integral role in regulating mental and physical fatigue after exercise.

Exhaustive exercise results in the branched-chain amino acids (BCAAs; leucine, isoleucine, and valine) being taken up by the skeletal muscle, leading to reductions in the plasma concentration. In the past 2 decades, numerous studies have shown the advantageous effects of BCAA supplementation on acute and chronic exercise performance, including improvements in time to exhaustion and strength [11,12]. Furthermore, studies in animal models suggest that BCAAs decrease the transport of tryptophan and its metabolites into the CNS because BCAAs and tryptophan compete for the same carrier system. In addition, it has been shown that the depletion of plasma BCAAs after dialysis is highly associated with mental fatigue [13]. In animal models, BCAAs attenuate changes in skeletal muscle PGC-1α caused by acute exercise by approximately 50% [14]. Although currently unexplored, these data suggest that when combined with exercise, BCAA supplementation may have the ability to modify mental and physical fatigue by modulating the tryptophan pathway.

### Objectives

This pilot study will examine the influence of systemic and skeletal muscle tryptophan metabolism on mental and physical fatigue after exercise training with and without BCAA supplementation in fatigued older adults. Our central hypothesis is that 8 weeks of daily BCAA supplementation added to 3-times-per-week exercise training will increase systemic and skeletal muscle expression of KAT, shifting kynurenine metabolism toward the enhanced synthesis of kynurenic acid, thereby reducing mental and physical fatigue.

## Methods

### Study Design and Overview

We propose a randomized, double-blind, placebo-controlled pilot study. The study protocol follows the SPIRIT (Standard Protocol Items: Recommendations for Interventional Trials) guidelines [15]. Any deviations from the protocol, breaches of confidentiality, and adverse events will be reported to the institutional review board (IRB) and data safety monitoring board (DSMB) according to local policies. In addition, the DSMB will evaluate study materials and review the collected data to ensure data security, integrity, and quality assurance. The study is registered with ClinicalTrials.gov (NCT05484661). As shown in Textbox 1, the experimental design involves 5 phases, completed over 3 to 4 months. In phase 1, we will recruit, screen, and enroll 30 participants (n=15, 50% per group). In phase 2, all participants will undergo baseline testing (described in the following subsections). In phase 3, participants will be randomly assigned to the exercise+placebo group or exercise+BCAA group, and we will test the acute effects of BCAA intake on fatigue and changes in the biomarkers of tryptophan metabolism and inflammation through a blood draw that will be performed after a 12-hour fast and 30 minutes after consuming either BCAA or a placebo. Phase 1 to 3 data will be collected at the Sam and Ann Barshop Institute for Longevity and Aging Studies of the University of Texas Health Science Center at San Antonio. Study phase 4 is the intervention and will consist of exercise training 3 times per week over 8 weeks with or without daily BCAA intake. To test the chronic effects of fatigue, participants will repeat all phase-2 tests at the end of the intervention (phase 5). Intervention staff and participants will be blinded to supplement allocation. Participants will receive either the BCAA or a placebo, both in powder form. Only study team members without direct participant interaction will know supplement allocation.

**Study design and timeline.**

**Week –4 (phase 1)**

- Recruitment
- Screening
- Enrollment (n=30)
- Consent

**Week –2 (phase 2)**

- Baseline tests and assessments
- Strength and endurance
- Blood draws
- Anthropometrics
- Muscle biopsies
- Questionnaires

**Week 0 (phase 3)**

- Randomization (1:1)
- Acute fatigue test

**Week 1 (phase 4)**

- Exercise+branched-chain amino acid group (n=15)
- Exercise+placebo group (n=15)
- 8-wk intervention

**Week 9 (phase 5)**

- Postintervention assessments (repeat assessments outlined in phase 2)

### Phase 1: Participant Recruitment, Screening, and Enrollment

#### Participants and Inclusion and Exclusion Criteria

The study will recruit a total of 30 community-dwelling older adults from the San Antonio, Texas, area. The inclusion criteria are as follows: (1) fatigue (participants self-reporting ≥3 on a 1-10 scale), (2) aged 60 to 80 years, (3) lack of menses for at least 1 year for women, (4) BMI 20 to 40 kg/m^2^, and (5) untrained with regard to structured exercises training (≤2 times/wk). The exclusion criteria are as follows: (1) taking an anticoagulant medication (ie, heparin, apixaban, or rivaroxaban); (2) allergic to lidocaine; (3) neurologic, musculoskeletal, or other condition that limits the participant’s ability to complete study physical assessments; (4) active inflammatory, autoimmune, infectious, hepatic renal, gastrointestinal, malignant, or psychiatric disease; (5) cognitive impairment (Montreal Cognitive Assessment [MoCA] score <21); (6) consuming a special diet or protein or CAA supplements; and (7) uncontrolled depression (Center for Epidemiologic Studies Depression Scale [CES-D] score ≥16).

#### Recruitment

Recruitment methods may include electronic medical record and approved registry queries, posting study flyers at medical offices or senior centers, community engagement activities, and newspaper or web-based advertisements (including social media). Telephone prescreening before the clinic screening visit will be used to ensure that potential participants meet the inclusion and exclusion criteria for enrollment. Those who qualify preliminarily will be scheduled for a full in-clinic evaluation and will be sent an advance copy of the consent form.

#### Screening

We will conduct an in-clinic evaluation consisting of a medical history; physical examination; resting, seated blood pressure; weight; height; and electrocardiogram. A fasting blood profile for lipids as well as liver, renal, and hematological function will be performed.

#### Enrollment

Participants will be enrolled if they provide informed consent and pass all required screening assessments.

### Phase 2: Baseline Assessment Measures

Participants will complete questionnaires related to their mental fatigue, depression, quality of life, and physical fatigue (they will be asked to perform tests of strength and endurance; Textbox 2). In addition, participants will have blood draws and muscle biopsies to assess markers of inflammation or immune status, quantify amino acid and metabolite levels, and examine changes in muscle gene expression and intracellular signaling pathways (ie, PGC-1α and KAT).

**Baseline and postintervention assessment measures.**

**Laboratory tests**

- Blood draw: plasma, serum, and whole blood will be collected for the assessment of thyroid-stimulating hormone, complete blood count, and a comprehensive metabolic panel with liver function tests and lipid profile. Plasma will be processed for the quantitative assessment of branched-chain amino acids and kynurenine metabolites using liquid chromatography–mass spectrometry, whereas plasma C-reactive protein, interleukin-6, interleukin-1β, and tumor necrosis factor-α will be measured by enzyme-linked immunosorbent assay (ELISA). Immune function (immunoreactivity) will be measured in diluted whole blood stimulated in vitro with 0-1000 ng/mL lipopolysaccharide (LPS) or 0-1000 ng/mL dexamethasone in the presence of 100 ng/mL LPS. Secreted cytokines will be measured by ELISA.
- Skeletal muscle biopsy: vastus lateralis muscle biopsies of the dominant leg will be obtained with a Bergström needle with local anesthesia by a credentialed clinician after a 10- to 12-h fast. Muscle tissue will quantitate local inflammation and tryptophan metabolism markers.

**Anthropometric measurements**

- BMI: weight (kg) will be measured on a standard physician’s scale at baseline and after the intervention without shoes and with light clothing. Standing height (cm) will be measured using a stadiometer at screening. BMI will be calculated as weight (kg)/height (m^2^).
- Waist and hip circumference: minimum and maximum waist and hip circumference will be measured with a tape measure. Measurements will be taken by trained personnel and will follow the World Health Organization guidelines.
- Body composition: whole-body dual-energy x-ray absorptiometry will be performed to measure lean and fat body mass and to examine the effect of the intervention on body composition [10,16]. In participants who are overweight where portions of the body fall outside the scan area, a half-body scan of the right side is carried out and a contralateral estimated performed [17].

**Quantitative assessments**

- Energy intake and expenditure
- Automated Self-Administered 24-Hour Dietary Assessment (ASA24): this validated web-based tool developed by the National Cancer Institute that enables multiple, automatically coded, and self-administered 24-h diet recalls will be used to calculate energy intake. The ASA24 will be used to calculate the Healthy Eating Index (HEI). HEI scores range from 0 to 100, with higher scores indicating better adherence to the Dietary Guidelines for Americans [18,19].
- Activity monitor (ActiGraph): ActiGraph GT9X Link accelerometers will be used to monitor total daily activity energy expenditure (kcal/d). Participants will wear an accelerometer around the right hip continuously for 7 d, except when sleeping, bathing, or swimming. In addition, participants will subjectively log their activity on the days that the accelerometer is worn.
- Short HEI (sHEI): The sHEI will be used to measure diet quality and assess how well dietary patterns align with the key recommendations of the Dietary Guidelines for Americans [20].
- Patient-Reported Outcomes Measurement Information System: this 57-item validated questionnaire will be used to evaluate and monitor 7 health domains (depression, anxiety, physical function, pain, sleep, fatigue, and social health) in adults [21].
- Mental fatigue (self-report measures)
  Fatigue Assessment Scale: this 10-item validated fatigue measure scale will be used to evaluate the severity of fatigue and its impact on daily living [22].
  Center for Epidemiologic Studies Depression Scale (CES-D): this 20-item screening test will be used to assess depression and depressive disorder. The CES-D measures symptoms defined by the American Psychiatric Association’s Diagnostic and Statistical Manual of Mental Disorders, Fifth Edition for a major depressive episode [23].
  Minnesota Leisure Time Physical Activity Questionnaire (MLTQ): this validated questionnaire will be used to measure physical activity (duration and frequency) and energy expenditure during leisure time physical activity [24].
  Insomnia Severity Index: this 7-item questionnaire will be used to assess the nature, severity, and impact of insomnia and monitor treatment response in adults [25].
  Visual analog scale: this validated psychometric measuring instrument will be used to measure global pain, fatigue, and quality of life, with scores for each measure ranging from 0 to 100 mm and higher scores indicating greater intensity of pain, tiredness, and fatigue as well as better quality of life [26,27].
- Physical fatigue (strength and endurance)
  Isometric handgrip: handgrip strength of both arms will be assessed using a handheld dynamometer. Measures will be taken in triplicate to take the average of the 3 measures [28].
  Strength: Knee extension and chest press strength will be measured by a standardized 1-repetition maximum (1-RM) protocol that includes 4 to 6 trials with rest periods. Muscle endurance will be assessed by asking the participant to perform repetitions of the leg extension and chest press exercises at 60% 1-RM to volitional fatigue, with the number of repetitions recorded [29].
  Chair rise: the chair rise test will be used to assess functional lower extremity strength. Chair stand number will be recorded as the number of chair stands completed as quickly as possible in 30 s with arms folded across the chest [30]. Assessment will be administered following the Stopping Elderly Accidents, Deaths, & Injuries protocol developed by the Centers for Disease Control and Prevention. Furthermore, the amount of time (s) required to sit and stand 5 times in succession will be recorded [31].
  Cardiopulmonary exercise test (CPET): a resting electrocardiogram (ECG) and blood pressure reading will be collected before the test. Open circuit spirometry will be performed during a CPET using a customized protocol to evaluate the cardiopulmonary response (ie, peak oxygen uptake [VO2peak] and ventilatory threshold) to strenuous exertion under clinical supervision and continuous vital sign and ECG monitoring according to the participant’s tolerance, perceived exertion, and gait stability. The test will be reviewed and participants cleared to participate by a research clinician [32].
  400-meter walk: the 400-meter walk test will used to measure walking ability and endurance [33]. The total time (s) taken to complete 400 m, maximal heart rate, and perceived exertion (rated on a scale ranging from 0 to 10, with 10 indicating maximum intensity) will be assessed.
- Frailty: frailty will be classified using the Fried phenotype criteria [34]: (1) self-reported unintentional weight loss of ≥10 lb (≥4.5 kg) in the past year, (2) self-reported exhaustion, (3) low energy expenditure measured using the MLTQ to assess physical activity (duration and frequency), (4) weakness measured via grip strength using a handheld dynamometer in the dominant hand, and (5) slow walking speed measured by 10-foot walk test. A frailty score will be calculated as the number (0-5) of frailty characteristics present. Those with ≥3 of these 5 characteristics are categorized as frail, those with 1 or 2 are categorized as prefrail, and those with none are categorized as nonfrail [35].

### Phase 3: Randomization

Participants will be randomized 1:1 to the exercise+placebo group or exercise+BCAA group. At this visit, participants will have resting vital signs assessed and 10- to 12-hour fasting blood draw performed. After the fasting blood draw, participants will consume (blinded) either 100 mg/kg of the BCAA or the maltodextrin (depending upon group allocation) and then will rest for 30 minutes. Immediately after the 30-minute rest, a 400-meter walk will be completed (as outlined in Textbox 2). A second blood draw will be performed within 3 to 5 minutes of completing the 400-meter walk.

### Phase 4: Exercise+Placebo or Exercise+BCAA Intervention

#### Exercise

Participants will attend in-person or web-based exercise sessions 3 times per week for 8 weeks. The exercise training protocol is designed to provide a high-volume, moderate-intensity, whole-body stimulus. The exercise will be prescribed by an exercise physiologist via provided heart rate (HR) monitors. Aerobic exercise training will start conservatively with a goal of 30 minutes total duration at 40% to 50% of maximal HR reserve determined according to the formula developed by Karvonen et al [36]:

Training HR = %(HRmax − HRrest) + HRrest **(1)**

HRmax is defined as peak HR assessed during a peak cardiopulmonary exercise test (described in Textbox 2). Aerobic exercise will advance weekly, as tolerated, to a goal of 75% to 85% of HR reserve for 30 minutes. In addition, participants will perform resistance exercise using TheraBands. They will perform the chest press, knee extension, leg curl, row, and bicep curl (15 repetitions for 2 sets and to exhaustion on the third set) for 5 major muscle groups. Resistance will be gradually increased to account for strength gains when participants are able to complete 20 repetitions on the third set.

#### BCAA or Placebo

##### Overview

This study will use over-the-counter dietary supplement powders. The BCAA supplement is manufactured and distributed by Naked Nutrition. One scoop (5 g) contains 2500 mg of L-leucine, 1250 mg of L-isoleucine, and 1250 mg of L-valine and sunflower lecithin. The placebo (maltodextrin) is manufactured and distributed by Nutricost. One scoop (15 g) contains 14 g of a complex carbohydrate (maltodextrin).

##### Labeling and Distribution of BCAA and Placebo Supplementation

Following double-blinded procedures, the study team will receive the BCAA or placebo supplements in prepackaged containers for the use of individual participants from the on-site clinical pharmacy. The pharmacy will maintain inventory; ensure appropriate temperature control; label each individual participant container with study and participant ID; and be responsible for storage, inventory, and destruction, following pharmacy protocol.

##### BCAA and Placebo Consumption

Participants will be randomly assigned to 8 weeks of BCAA or placebo supplementation. Participants will be instructed to consume either 7 to 10 g (100 mg/kg body weight) of BCAA supplement or placebo daily. The powders, identical in color, will be distributed in a double-blind fashion and dissolved in 400 mL of water and 2.5 g of a calorie-free water flavor enhancer (ie, Crystal Light). The supplement to be consumed on nonexercise days will be in prepackaged containers and provided to participants along with water flavor enhancers on a weekly basis. On nonexercise days, participants will be instructed to take the supplement between 9 AM and 10 AM each day and to log each intake on a provided spreadsheet. On exercise days, the supplement will be consumed immediately after finishing exercise training while being monitored by study staff. The distribution and consumption of the supplement will be logged by study staff. In addition, participants will be asked about supplement intake at every exercise session to track compliance. The assessment of side effects will be performed at every exercise session.

### Phase 5: Postintervention Measures

After the intervention, participants will repeat all baseline (phase 2) assessment measures and qualitative assessments (Textbox 2).

### Outcomes and Analysis

An important goal of statistical analyses is to determine effect sizes to make certain that future studies evolving from the current work are adequately powered. Although data do not exist to provide more precise effect sizes for this experiment, based upon previous studies [37], a mean decrease of 0.64 (SD 0.19) µmol/L in kynurenine concentration was observed after exercise training. The anticipated additive effect of BCAA supplementation is 0.25 µmol/L. Assuming an SD of 0.25 µmol/L, 12 participants per group are necessary to achieve at least 80% power with a significance level of 5%. To allow for a 20% dropout rate, we will recruit a total of 30 participants with the intent of having 24 complete the study.

In light of the small sample size, 2 separate independent samples 2-tailed *t* tests will be used for each outcome measure (ie, mental fatigue and physical fatigue) to compare changes between the BCAA and placebo groups: (1) before and after phase 3 acute exercise (400-meter walk acute fatigue test) and (2) before and after chronic (8-wk changes from phase 2 to phase 5) exercise training. We will use intention-to-treat principles per CONSORT (Consolidated Standards of Reporting Trials) guidelines to avoid the effects of attrition, which may break the random assignment to the treatment groups in a study. All statistical tests will be 2-sided and unadjusted for multiple comparisons.

### Participant Safety and Minimizing Potential Risk

#### Overview

The risks from participating in the research study are mild to moderate and involve some slight temporary discomforts. Procedures that involve a degree of risk include completing the questionnaires, exercise training, functional assessment, BCAA or placebo supplementation (maltodextrin), muscle biopsy, blood draws, and dual-energy x-ray absorptiometry (DXA) scans. Halting the study will be at the discretion of the study clinical staff and DSMB. There is no known risk of stopping the study supplements early or abruptly.

#### Questionnaires

The questionnaires are time consuming but pose minimal risk.

#### Exercise Training and Functional Assessments

The physical activity requirement for this study is associated with shortness of breath, muscle soreness, and strains, along with the risk of cardiovascular complications such as chest pain, heart attack, or sudden death. To offset this minimal risk, all participants will undergo prescreening with medical examinations before exercise. In addition, an exercise physiologist or other medical professional will be present for all exercise sessions. There is also a minimal risk of falling during the fatigue assessment walking tests. A standby aide will always be present, and sufficient periods or rest will be provided to minimize risk.

#### Supplementation

The risks from consuming the BCAA and maltodextrin are expected to be minimal and may include gastrointestinal distress, such as cramps and gas. Participants will be monitored at each exercise session, and any unanticipated or serious side effects will be reported immediately to the study clinical staff, IRB, and DSMB.

#### Muscle Biopsy

Mild to moderate discomfort is likely but not serious and may include a sensation of pressure or thumping, cramping, pain, soreness, and bleeding. The pain is typically mild to moderate and lasts 5 to 10 seconds. The pain usually stops when the needle is removed but muscle soreness could last for a few days. Ice packs are applied to the biopsy site to minimize discomfort.

#### Blood Draw

The risks associated with blood sampling include discomfort, bruising, swelling, fainting, and possible infection at the site of sampling. This is minimized by having skilled professionals perform the blood draw.

#### DXA Scans

Participants will be exposed to a minor dose of ionizing radiation (total 0.6 mrem/scan) during the DXA scans at phase 2 and phase 5 [16], which is well below the dose of a standard chest x-ray (10 mrem; United States Environmental Protection Agency Radiation Sources and Doses [38]).

### Data Management

All research records created will be held strictly confidential. A study number rather than the participant’s name will be used on study records whenever possible. Electronic data will be password protected and placed on the secure local area network (REDCap [Research Electronic Data Capture; Vanderbilt University]; hosted at the University of Texas Health Science Center San Antonio]). Randomization codes will be created at random and kept electronically by a study team member not directly involved with participants (ie, the statistician). We anticipate that blinding will only be broken before the completion of the study if requested by the DSMB owing to safety concerns. Unblinding before study completion will be reported to the IRB and DSMB.

### Ethical Considerations

The study was approved by the local IRB (University of Texas Health Science Center at San Antonio; 20220493H) in accordance with the ethical standards of the responsible committees on human experimentation and the Declaration of Helsinki. Eligible participants will have the study explained and an in-person IRB-approved informed consent will be obtained from each participant. Participants may choose to leave or opt out the study at any time during the study without penalty or loss of benefits they are entitled. All data and biospecimens will be deidentified. Participants will receive US $50.00 compensation after completion of the 2 testing phases (phase 2 and phase 5) for a total of US $100.00 for completion of the entire study.

### Dissemination

The results of this study will be made available to health care professionals and the public through the National Library of Medicine PubMed Central website within 1 year after the date of publication. In addition, the findings of this study will be presented at scientific meetings. The study investigators will be responsible for writing all publications and will not use the services of professional writers.

## Results

The study was funded in March 2022, with a projected data collection period lasting from January 2023 through December 2023. The screening and enrollment of participants for phase 1 began in December 2022. Data collection began in January 2023 and is projected to be completed at the end of November 2023. As of August 2023, a total of 24 participants have been recruited and enrolled in the study. Data analysis and results are aimed to be published in early 2024.

## Discussion

### Summary

The purpose of this study is to examine whether the addition of a BCAA to acute and chronic exercise can reduce mental and physical fatigue using a pilot randomized, double-blind, placebo-controlled trial. This study will also address whether clinical outcomes are modulated by changes in circulating and skeletal muscle tryptophan metabolism because this may identify potential targets for future investigation. We anticipate that 8 weeks of a BCAA added to exercise will increase the expression of KAT, shifting kynurenine metabolism toward the enhanced synthesis of kynurenic acid, thereby reducing fatigue.

Fatigue is one of the most common idiopathic complaints in older adults [39], with up to 50% of older adults aged 65 years [40] and 75% of those aged >85 years [41] self-reporting symptoms. Studies show that both mental fatigue and physical fatigue are associated with accelerated decline in physical function and cognition [42], which are strong predictors for the loss of independence in community-dwelling older adults [43]. Although fatigue can significantly affect an individual’s quality of life, social well-being, and livelihood [44], its etiology remains unknown, and effective treatment strategies remain elusive.

Tryptophan is the precursor of serotonin and kynurenine, and accumulating evidence suggests that kynurenine metabolic pathways are associated with mental and physical fatigue [45]. A recent study demonstrated that the uptake of peripheral kynurenine into the brain of mice increased kynurenic acid production and induced mental fatigue [45]. This supports previous evidence that has shown that the ingestion of tryptophan by humans can also induce fatigue [45,46]. These data suggest that the tryptophan metabolism pathway may play an integral role in regulating mental and physical fatigue.

Exercise has been associated as a low-cost strategy for improving symptoms linked to fatigue [47]; however, symptoms are not eliminated. A recent randomized controlled trial has shown that exercise training can reduce the incidence of moderate to severe mental and physical fatigue by 30% to 60%; however, 82% of the participants continued to report mild fatigue after the intervention [9]. Animal models suggest that the reduction of fatigue from exercise is mediated by skeletal muscle PGC-1α, which shifts tryptophan metabolism [9] and therefore may influence fatigue. A recent study in older adults found that exercise training significantly increased skeletal muscle PGC-1α gene expression [10]. In addition, it has been shown that sedentary fatigued older adults had decreased concentration and suppression of PGC-1α in muscle tissue compared with nonfatigued older adults [48].

BCAAs play a critical role in the regulation of multiple physiological functions, including nutrient metabolism, energy homeostasis, immunity, intestinal health, protein synthesis, and aging [49]. Evidence suggests that BCAA supplementation can be beneficial during exercise, with studies showing improved ratings of perceived exertions and mental performance [49,50]. The consumption of BCAAs causes a rapid increase in serum concentration and competes with tryptophan for entry into the CNS [51]. In addition, BCAAs have been shown to attenuate changes in skeletal muscle PGC-1α caused by exercise [11,12]. Although currently unexplored, data suggest that, when combined with exercise, BCAA supplementation may have the ability to modify mental and physical fatigue by modulating the tryptophan pathway. Therefore, this study will fill a knowledge gap by determining whether changes in tryptophan metabolism occur after exercise with and without BCAA supplementation and relate to reductions in mental and physical fatigue, because this may identify potential targets for future lifestyle and pharmacologic investigation.

The identification of BCAA plus exercise as being superior to exercise alone could have clinical implications for older adults to improve cardiometabolic and functional risk. By reducing fatigue, older adults may be able to engage in longer periods of physical activity, thereby reducing weight gain common to older adults and improving the mental and physical quality of life.

### Limitations

A randomized controlled trial is generally accepted as the gold standard for investigating causal relationships between an intervention and outcomes; however, despite its strength, this study design has potential limitations that should be considered. The small sample size increases the possibility of a type II error, which decreases the power of the comparison. In addition, the potential for heterogeneity between the intervention and control groups could result in false-positive results [52]. The use of self-report measures for some clinical outcomes limits any conclusion from being drawn about cause and effect and limits the generalizability of the results. Furthermore, participants could have low adherence to the study protocol although study has been designed to mitigate risk.

### Future Direction

This pilot will provide preliminary evidence for larger grant applications to examine the combined effects of BCAA supplementation and exercise on mental and physical fatigue. In addition, based on the findings from this project, future clinical studies may address how other nutrient-rich foods or supplements or weight loss could further alter the response to BCAA and exercise. Furthermore, there are numerous other important clinical outcomes related to fatigue that could be examined as influenced by BCAA and exercise (ie, muscle quality, fall risk, and cognitive functioning). The discovery that kynurenine concentrations are associated with fatigue and are responsive to BCAA supplementation during exercise training could have important implications for the development of future interventions, both lifestyle and pharmacologic, to treat fatigue in older adults.

### Conclusions

Through BCAA supplementation during exercise, the clinical research proposed herein has the potential to provide insight into whether BCAA supplementation can effectively reduce mental and physical fatigue when combined with acute and chronic exercise and whether these improvements are related to alterations in tryptophan metabolism in fatigued older adults. This could have important implications for prescribing exercise in older adults and the development of future interventions, both lifestyle and pharmacologic, to treat fatigue in older adults.

## Appendix

Multimedia Appendix 1 Peer review report from the National Institute on Aging (National Institutes of Health, USA).

## Abbreviations

BCAA: branched-chain amino acid
CES-D: Center for Epidemiologic Studies Depression Scale
CNS: central nervous system
CONSORT: Consolidated Standards of Reporting Trials
DSMB: data safety monitoring board
DXA: dual-energy x-ray absorptiometry
HR: heart rate
IRB: institutional review board
KAT: kynurenine aminotransferase
MoCA: Montreal Cognitive Assessment
PGC-1α: peroxisome proliferator-activated receptor γ coactivator 1α
REDCap: Research Electronic Data Capture
SPIRIT: Standard Protocol Items: Recommendations for Interventional Trials

## Data Availability

A deidentified final data set underlying all publications resulting from the proposed research will be shared on request pursuant to a data use agreement, appropriately limiting the use of the data set and prohibiting the recipient from identifying or reidentifying (or taking steps to identify or reidentify) any individual whose data are included in the data set.
