# Supplementary material for: The Influence of Branched-Chain Amino Acid Supplementation on Fatigue and Tryptophan Metabolism After Acute and Chronic Exercise in Older Adults: Protocol for a Pilot Randomized Controlled Trial
Source: JMIR Res Protoc. 2023 Nov 1;12:e52199. doi: 10.2196/52199 (PMC10652194; doi:10.2196/52199)
Supplement: Multimedia Appendix 1 [file resprot_v12i1e52199_app1.pdf]

# Application: PESC2021--0000000015

Monica Serra - serram@uthscsa.edu
Pepper Center (OAIC) Pilot Projects

## Application Review Round I for:

**Completed** - May 29 2021
**Score:** 2/9

# Review Task Form

### Project PI (Principal Investigator)

| | |
|---|---|
| First name | Monica |
| Middle | (No response) |
| Last name | Serra |

### Reviewer

| | |
|---|---|
| First Name | |
| Last Name | |

### Review comments

**Significance, novelty, innovation:**

Strengths:

The study investigates two factors important for aging – 1) how to address fatigue in older adults (physical and mental) 2) whether acute or chronic exercise are the mechanistic factors.

Decreases in fatigue can lead to many heath promoting outcomes

Weaknesses:

Limited to dietary intake – study does not account for individualistic factors/cognitions that ultimately affect behavior & perceptions – however adding another layer like this would be too much scope for a pilot study

o (score 1)

**Strength of the study protocol (rationale, design, feasibility, preliminary data [if available]):**

Strengths:

The overall design is excellent – randomization the placebo versus the branch chain amino acid supplement; the recruitment, screening, treatment of data are well thought out and communicated.

Overall well thought out, scientifically valid research plan.

Weaknesses:

Enthusiasm is dampened by an overall lack of detail in why specific choices were made – this includes not having citations to support many of the decisions. Some (but not all) examples follow:

o Why 8 week as the trial period

o “supplement will be consumed right after training – why not right before – why this time

o How is food and drink intake documented exactly

o Which accelerometer is used (Fitbit?)

o Metronome set at 30 (beats per minute?)

o What is the effect size in the power calculation

o The integrity of the data is put at risk with what can be considered heavy demand on participant self-reporting and recording

(score 3).

**Qualifications of the investigator(s) (PI, other team members, career development potential):**

Strengths:

The research team is more than qualified. Dr. Serra’s own expertise is transdisciplinary (exercise, diet, again, stress etc etc)

The research coordinator is a very solid asset to have on this project.

Weaknesses:

None noted.

(score 1)

---

**Interdisciplinary collaboration and pre-clinical/clinical research on aging intervention:**

Strengths:

The PI has diet/exercise/counseling/again expertise and has brought in a solid investigator, pharmacist, Dr. O’Connor.

Weaknesses:

Only one collaborator.

(score 2)

---

**Likelihood of future NIH or other competitive external funding:**

Strengths:

Competitive – if the hypotheses are accurate then increased probability of securing external funding.

Weaknesses:

None noted.

(Score 1)

---

**Use and leveraging of OAIC Core facilities:**

Strengths:

The Clinical Research and Pharmacology Core (RC 2 core is committed as is Dept of Population Health Sciences (Dr. Gelfond) – providing (statistical and bioinformatics support)

Weaknesses:

None noted.

(Score 1)

---

**Brief summary of strengths, weaknesses and overall merit:**

Strengths

o The study investigates two factors important for aging – 1) how to address fatigue in older adults (physical and mental) 2) whether acute or chronic exercise are the mechanistic factors
o Decreasing in fatigue can lead to many heath promoting outcomes.
o The PI has forged a strong team – the triad of Dr.'Connort, Dr. Gelfond, Dr. Serra and the research coordinator chosen has morphed into what is anticipated to be very knowledgeable, proactive, quick to respond team.
o Clinical Research and Pharmacology Core (RC 2 core is committed as is Dept of Population Health Sciences (Dr. Gelfond) – providing (statistical and bioinformatics support)
o The study is innovative and important addressing an important need for better understanding 'fatigue" as a challenge to health-related quality of life.

Weaknesses:
o Enthusiasm for this important study is dampened due details being left out in the proposal. This focuses more on why references weren't cited; and, details of research plan are not explained

an excellent scientific pilot, investigating an important area ; with easily addressable minor issues related to level of detail provided in the research plan
overall score – 1.5 (average of above)

**Do you have concerns about the budget or protection of human subjects or vertebrate animals, if applicable? If yes, to any, please comment:**

Yes
$5,000 for biostatistics support – think that should be broken down in to more specifics – how arrived at this $ number and justification.

(minor issue though)

**Priority Score – Whole number between 1 – 9, with 1 being exceptional, 5 – average, 9 - poor**

2

## Application Review Round I for:

**Completed** - Jun 2 2021
**Score:** 2/9

# Review Task Form

**Project PI (Principal Investigator)**

| | |
|---|---|
| First name | Monica |
| Middle | (No response) |
| Last name | Serra |

**Reviewer**

| | |
|---|---|
| First Name | |
| Last Name | |

**Review comments**

**Significance, novelty, innovation:**

This is a significant, novel, and innovative pilot study designed to compare exercise plus placebo (EX+PLA) to exercise plus branched-chain amino acids (BCAAs; (EX+BCAA) on changes in mental fatigue, in association with changes in systemic and skeletal muscle tryptophan metabolism. It is difficult enough to motivate individuals to exercise. The citation that in a study conducted by Dr. Serra 82% of subjects continued to report mild fatigue after the intervention is concerning. A supplement including the 3 amino acids leucine, isoleucine and valine that decreased fatigue following exercise would be an important finding and contribution both to the scientific literature and the health of the nation.

**Strength of the study protocol (rationale, design, feasibility, preliminary data [if available]):**

Dr. Serra is proposing a well-designed RCT testing EX+PLA to EX+BCAA in 24 fatigued older adults. The inclusion/exclusion criteria are well thought out (e. g., recruiting participants with a subjective rating of fatigue >3 on a VAS who are sedentary) and should maximize the difference between the groups, if there is a difference. Dr. Serra might consider assessments other than the Beck Depression Inventory (I didn't see funds budgeted to cover the expense of administering the Beck) and the Pittsburg Sleep Quality Index (which is difficult to score and has a constrained variance, i. e., 0-5 = normal sleep while 5-21 indicates poor sleep). Likewise, the SF-36 Quality of Life scale is difficult to score and while acceptable as a snapshot of overall health compared to a population mean, is not particularly responsive to change especially to an 8-week exercise and supplement intervention. These are minor considerations, however, that Dr. Serra can consider prior to starting her project should it be funded. It's not completely clear to me who will be performing the study procedures and testing, i. e., the exercise intervention, preparation and dispensing of the PLA and BCAA, blood draws, medical history intake, the Cardiopulmonary Exercise Test (CPET), muscle biopsies, and laboratory testing. Two research staff members are budgeted for, a study coordinator (Kelseia Hertzog) and a laboratory technician student (Grace Porter). Dr. Serra currently works with Ms. Hertzog on another of her projects and Ms. Porter is a graduate student in Dr. O'Connor's lab. While it is reasonable that these two individuals do much of the work, I don't believe that they are credentialed do all the study activities proposed.

**Qualifications of the investigator(s) (PI, other team members, career development potential):**

Dr. Serra is a well-prepared clinician and researcher with expertise in both nutrition and exercise science. Very few individuals are prepared in both disciplines although the advantages of considering both nutrition and exercise in the design of studies is obvious. Dr. Serra joined the faculty of UTHSCSA in 2019 and is building her research career here. She should be supported in this effort. I suspect that she has more mentors and collaborators at UTHSCSA and the Pepper Center whose roles could not be fully described in a brief pilot project application.

**Interdisciplinary collaboration and pre-clinical/clinical research on aging intervention:**

Dr. Serra’s collaborator, Dr. Jason O’Connor, is an Associate Professor in the Department of Pharmacology and has several ongoing projects that will be synergistic with Dr. Serra’s work. Dr. Serra also proposes to collaborate with Dr. Sara Espinoza, Co-Leader, Clinical Research and Pharmacology Core. It’s unclear if Dr. Jonathan Gelfond is affiliated with the Trail Design and Integrative Informatics Core or if he is providing biostatistical support based on his UTHSCSA faculty position in the Department of Population Health Sciences.

---

**Likelihood of future NIH or other competitive external funding:**

Excellent with the preliminary data this application proposes to collect.

---

**Use and leveraging of OAIC Core facilities:**

From the web, I identified the Older Americans Independence Center (OAIC) Cores to be Leadership and Administrative, Research Education, Pilot and Exploratory Studies, Pre-Clinical Research, Clinical Research and Pharmacology, Trail Design and Integrative Informatics, and the South Texas Aging Registry and Repository. As best I can discern, Dr. Serra will use the services of 2, perhaps 3 of these cores, i. e., Clinical Research and Pharmacology (Sara Espinoza, MD, MSc, Co-Leader, Clinical Research and Pharmacology Core), the South Texas Aging Registry and Repository (for recruitment), and potentially the Trial Design and Integrative Informatics (Jonathan Gelfond). Seemingly another 2 Cores could support Dr. Serra, the Leadership and Administrative, Pilot and Exploratory Studies Cores.

---

**Brief summary of strengths, weaknesses and overall merit:**

Strengths: Innovative idea, opportunity to make a major impact on the health of older individuals, well-designed randomized pilot, important developmental work that will build upon and expand Dr. Serra's ongoing program of research.

Weaknesses: The space limitations of the application did not allow a full description of who will be doing all the study procedures and how the Older Americans Independence Center (OAIC) Cores can best assist Dr. Serra in the conduct of this project.

**Do you have concerns about the budget or protection of human subjects or vertebrate animals, if applicable? If yes, to any, please comment:**

No concerns.

**Priority Score – Whole number between 1 – 9, with 1 being exceptional, 5 – average, 9 - poor**

2

## Application Review Round I for:

**Completed** - Jun 5 2021
**Score:** 7/9

# Review Task Form

**Project PI (Principal Investigator)**

| | |
|---|---|
| First name | Monica |
| Middle | (No response) |
| Last name | Serra |

**Reviewer**

| | |
|---|---|
| First Name | |
| Last Name | |

**Review comments**

**Significance, novelty, innovation:**

The hypothesis is interesting. I don't the applicant really sells the scientific premise. I am unsure how big a problem "fatigue" really in older people undergoing exercise training except that the applicant says it is. I worry about the measure of mental fatigue proposed and whether it is sufficiently sensitive to change. Further concern about the measures of "physical fatigue" as they all are somewhat overlapping but yet also have specificity with the impairment that may drive the fatigue.

**Strength of the study protocol (rationale, design, feasibility, preliminary data [if available]):**

I would just say that the proposed trial really seems overly ambitious for a pilot.
The rationale for the modes of exercise training seems very generic. I don't think its feasible to complete in one year. The rationale for the dose of BCAAs is not provided. There really is no preliminary data. I would want to see more development of the intervention rationale and the selection of outcomes to feel comfortable about this going forward.

**Qualifications of the investigator(s) (PI, other team members, career development potential):**

PI and research team are capable of conducting the proposed studies. Career development plan was not robust.

**Interdisciplinary collaboration and pre-clinical/clinical research on aging intervention:**

The premise for this as written is pretty weak. I worry about the mental outcome really being responsive. The proposed molecular studies may be good and could provide preliminary data.

**Likelihood of future NIH or other competitive external funding:**

A lot would have to come together for this to lead to extramural funding in the short term.

**Use and leveraging of OAIC Core facilities:**

Core use seems reasonable.

**Brief summary of strengths, weaknesses and overall merit:**

Novel hypothesis.

Weak scientific premise.

Overly ambitious.

Intervention not well justified.

Should focus on one aspect of this project.

**Do you have concerns about the budget or protection of human subjects or vertebrate animals, if applicable? If yes, to any, please comment:**

I can't see how this can be accomplished on 50K.

No really human subject concerns. I am assuming that muscle biopsies are obtained at baseline and follow-up although that is not exactly stated.

**Priority Score – Whole number between 1 – 9, with 1 being exceptional, 5 – average, 9 - poor**

7
